# Aqueous Humour Ofloxacin Concentration after Topical Instillation in Patients with Dry Eye Disease

**DOI:** 10.3390/medicina58081031

**Published:** 2022-08-01

**Authors:** Konstantinos Kagkelaris, Mohamed A. El Mubarak, Panagiotis Plotas, Dimitris Panaretos, George D. Panayiotakopoulos, Gregory B. Sivolapenko, Constantinos D. Georgakopoulos

**Affiliations:** 1 Department of Ophthalmology, School of Medicine, University of Patras, 26504 Patras, Greece; cgeorg@upatras.gr; 2 Pharmacology Laboratory, School of Medicine, University of Patras, 26504 Patras, Greece; gpanayiotakopoulos@hotmail.com; 3 Laboratory of Pharmacokinetics, Department of Pharmacy, University of Patras, 26504 Patras, Greece; sheikho@upatras.gr (M.A.E.M.); gsivolap@upatras.gr (G.B.S.); 4 Lab Primary Health Care, School of Health Rehabilitation Sciences, University of Patras, 26504 Patras, Greece; pplotas@upatras.gr; 5 Laboratory of Clinical Pharmacology, School of Medicine, Aristotle University of Thessaloniki, 54124 Thessaloniki, Greece; dimitrispanaretos@hotmail.com

**Keywords:** dry eye disease, ofloxacin, aqueous humor concentration, topical instillation

## Abstract

*Background and Objectives*: A prospective, randomized clinical trial was conducted to evaluate the concentration of ofloxacin in the aqueous humour (AqH) of patients suffering from dry eye disease (DED) after topical instillation. *Materials and Methods*: Ninety-one (91) cataract patients scheduled for phacoemulsification were categorized into three groups according to DED severity. Group I (*n* = 17) was comprised of subjects without DED, patients in group II (*n* = 37) were evaluated as having non-severe DED, while group III (*n* = 37) consisted of patients suffering from severe DED. Preoperatively, patients received 4 drops of 0.3% of ofloxacin at 15 min intervals. One hour after the last instillation, aqueous samples were collected intraoperatively. *Results*: The median AqH concentration of ofloxacin in group I was 199.9 ng/mL (range 92.2–442.8 ng/mL), while in group II it was 530.5 ng/mL (range 283.7–1004.9 ng/mL), and 719.2 ng/mL (range 358.0–1512.4 ng/mL) in Group III, *p* < 0.001 (Kruskal-Wallis tests). Pairwise tests (two-tailed with Bonferroni corrections) between groups resulted in a *p*-value of 0.001 when group II was compared to group I and group III was compared to group I, and a *p*-value of 0.020 when group II was compared to group III. The severity of DED, across groups I, II, and III, and the levels of ofloxacin revealed a strong positive correlation (r = 0.639, *p* < 0.001). *Conclusions*: Ofloxacin concentration in the AqH after topical drop instillation may be affected by the degree of ocular surface inflammation in patients suffering from DED.

## 1. Introduction

Dry eye disease (DED) is a complex multifactorial disease of the ocular surface characterized by a loss of homeostasis of the tear film and accompanied by ocular symptoms, in which tear film instability and hyperosmolarity, ocular surface inflammation and damage, and neurosensory abnormalities play etiological roles [1]. It has globally evolved into a public health concern, and it shows increasing prevalence worldwide. Hyperosmolarity of the tear film initiates a vicious inflammatory cycle which finally leads to the disruption of the ocular surface. In particular, this leads to apoptosis of the conjunctival epithelial cells, cell membrane damage, a reduction in superficial corneal microvilli, corneal epithelia cell loss, and the disruption of intercellular tight junctions, resulting in corneal epithelial barrier dysfunction [2,3,4,5,6,7]. The resolution of the inflammation is controlled by immunoregulatory processes, but when these fail, the disease becomes amplified resulting in further damage to the ocular surface [3].

Ocular surface integrity is essential to attain clinically adequate drug levels following topical administration. Drug penetration into the anterior chamber (A/C) is mainly facilitated through the cornea (the corneal route), which acts as a rate-limiting barrier to drug diffusion due to its anatomy. Owning to its tight junctions and desmosomes, the epithelium poses greater resistance to permeation, mainly to hydrophilic drugs (molecular weight up to 300 Da) that cross it through the intercellular space between its cells (called the paracellular route). Hydrophobic molecules (molecular weight up to 600 Da), due to their ability to partition into the cell membranes, can transverse through the paracellular and intercellular pathways to access the underlying stroma. Microvilli of the apical surface extend the available area for drug absorption. The stromal layer acts as a barrier for hydrophobic molecules, whereas it allows the permeation of hydrophilic molecules. The endothelium also consists of cells linked with tight junctions, but it poses considerably less resistance to drug diffusion [8,9,10]. Instilled drugs are also absorbed through the conjunctiva. Its epithelium holds tight junctions to limit the permeability of high molecular weight drugs. Underlying lymphatics and blood vessels mainly remove the drug from the systemic circulation, reducing its bioavailability in the A/C [11,12].

Ofloxacin is a second-generation fluoroquinolone antibiotic that exhibits a wide range of antimicrobial activity and is commonly applied, pre- and/or post-operatively to avoid the deleterious effects of infectious endophthalmitis and numerous infectious ocular diseases [13,14]. Its molecular weight is 361.4 Da and its logK_ow_ is −0.48 (pH 7.2), rendering the molecule slightly hydrophilic in neutral pH, as the tears are [15,16]. In light of the increasing resistance of microbes, especially against older and widely used antibiotics, continuous surveillance is an essential measure to take in evidence-based therapeutic prophylaxis and the treatment of infectious diseases [17,18]. The choice of antibacterial agents needs to be adapted according to the offending bacteria and the site of infection. The drug’s ability to achieve a therapeutic level is affected by physiological and pathophysiological factors [19,20].

Given the inflammation and disruption of the ocular surface due to DED, the current study aimed to evaluate the levels of ofloxacin in the aqueous humour (AqH) of patients suffering from severe and non-severe DED after topical instillation.

## 2. Materials and Methods

Ninety-one Caucasian patients scheduled to undergo cataract surgery were studied. Patients were categorized into three groups according to DED severity [21]. Group I was comprised of 17 subjects without DED (8 male, 9 female, mean age ± SD of 71.2 ± 8.5 years), group II consisted of 37 patients (19 male, 18 female, mean age 71.5 ± 8.3 years) evaluated as having non-severe (mild/moderate) DED, and group III consisted of 37 patients (20 male, 17 female, mean age 77.3 ± 7.7 years) suffering from severe DED. Patients with chronic topical ocular treatment, the presence of exfoliation material within the anterior segment of the eye, pigment dispersion syndrome, corneal guttata, A/C angle < 20°, ocular pathologies other than cataract, abnormal eyelid function, diabetes mellitus, renal or hepatic failure, other local and systematic antibiotic treatment, allergy to fluoroquinolone antibiotics, or contact lens use were excluded from the study. The study was approved by the ethics committee of the University of Patras for human research and adhered to the tenets of the Declaration of Helsinki and the ICH-Good Clinical Practice guidelines. Written informed consent was obtained from all the study subjects before inclusion in the study (ClinicalTrials.gov ID: NCT05213156).

One day prior to cataract surgery, during standard cataract preoperative evaluation [22], all patients were clinically examined, and the severity of DED was determined in accordance with the algorithm proposed by Baudouin et al. [21]. A brief symptom-based assessment was delivered by scoring the Ocular Surface Disease Index (OSDI) questionnaire, and an evaluation of ocular surface damage was performed through corneal fluorescein staining using the Oxford scale.

Before surgery, patients received one drop of commercially available topical ofloxacin solution 0.3% (pH 6.4) at monodoses without preservatives (Oxatrex, Zwitter Pharmaceuticals, Athens, Greece), 4 times at 15 min intervals starting 2 h before surgery. The eye drops were applied to the inferior lower fornix. Patients who missed any of the four doses were excluded from the study. Before the administration of ofloxacin drops, patients were instilled one drop of commercially available phenylephrine 10% (Phenylephrine, Cooper S.A. Pharmaceuticals, Athens, Greece) and one drop of commercially available tropicamide 0.5% (Tropixal, DEMO S.A. Pharmaceuticals, Kryoneri, Greece) 3 times every 20 min. Aqueous humor was collected 1 h after the last administration, intraoperatively, at the beginning of cataract surgery.

Lids, eyelashes, the skin surrounding the globe, and eyelashes were cleaned with 5% povidone-iodine immediately before the operation. A paracentesis track was made with a 15° superblade. A 30 G cannula connected to a tuberculin syringe was inserted into the A/C, and approximately 50 μL of aqueous humor was withdrawn. AqH samples were collected in Eppendorf tubes and immediately placed on ice and protected from light until analysis. Within 1 h, all samples were frozen at −20 °C.

Ofloxacin concentrations were determined by HPLC–MS/MS, as described by El Mubarak et al. [23]. The HPLC–MS/MS analysis was performed according to Good Laboratory Practice guidelines and validated according to FDA and EMEA guidelines [24,25]. Concisely, AqH samples were vortexed, and 30 μL was reconstituted with 10 μL of internal standard (ciprofloxacin ≥ 98%, Merck, Darmstadt, Germany) and 300 μL of acetonitrile (HPLC grade, Sigma-Aldrich, Darmstadt, Germany). Solutions were vortexed for 1 min, centrifuged at 10,000× *g* for 5 min, and the supernatant was dried on a CentriVap cold trap concentrator (Labconco, Kansas City, MO, USA). Dried extracts were reconstituted in 1000 μL of 0.1% formic acid (HPLC grade, Sigma-Aldrich, Germany) in aqueous, filtered through RC 0.22 μm filters (Phenomenex, Torrance, CA, USA), and 50 μL was introduced into Waters HPLC system (Alliance HT 2795), which was equipped with a Micromass Quattro micro tandem MS system (Waters, Milford, MA, USA). A Synergi Hydro-RP column 100 × 2 mm, 4 μm, Proguard, 2 to 8 mm (Phenomenex, Washington, DC, USA) at 40 °C with a mobile phase consisting of 0.1% formic acid in aqueous (Solvent A) and acetonitrile (Solvent B), at a flow rate of 0.3 mL/min, were used. The MS system was set in positive ion mode to operate the electrospray ion source. Its settings were as follows: desolvation temperature, 450 °C; source temperature, 100 °C; desolvation gas flow, 500 L/h; collision gas (argon) flow, 50 L/h; capillary voltage, 3.5 kV, and the multiplier at 650 V; cone voltage, 33 V; collision energy, 16 eV. The selected transitions *m*/*z* (multiple reaction mode scans) for both ofloxacin and the internal standard were 362.1 > 318.1 and 332.1 > 288.2, correspondingly.

### Statistical Analysis

Statistical analysis was performed using SPSS software ver. 28.0 (IBM, Armonk, NY, USA). All data were checked for normality using the Kolmogorov–Smirnov test. G-Power 3.1.9 (Universität Kiel, Germany) was used to calculate the number of subjects that were required in each group to achieve a power level of 0.85. Initial descriptive statistics were undertaken. Data were presented as mean (± standard deviation [SD]) when variables had a parametric distribution, and as median with ranges or absolute frequency and percentage when the distribution was non-parametric. A comparison of the measurements among the three groups was performed using the Kruskal–Wallis test with pairwise tests (two-sided) with Bonferroni correction. The Bonferroni correction was applied to avoid the risk of committing a type I error. To be more precise, the alpha level of 0.05 was corrected for multiple comparisons involving the three outcome measures, resulting in an alpha level of 0.017. The correlation of DED severity with the concentration of ofloxacin in the AqH was evaluated using the Spearman correlation coefficient (two-sided). All statistical tests were performed at a 5% level of significance.

## 3. Results

**Figure 1:**
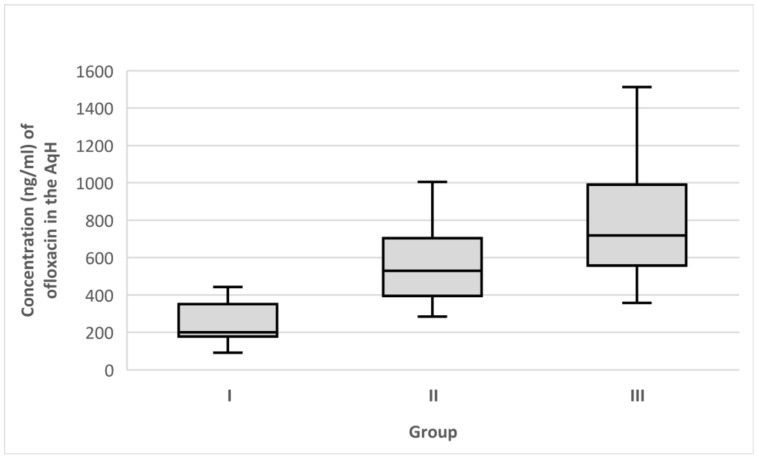
Median ofloxacin concentrations (ng/mL) in the AqH. Error bars represent the interquartile range. *p* < 0.001 (Kruskal–Wallis test) differences between group I and group II and groups I and III (pairwise comparisons). *p* = 0.02 differences between groups II and III (pairwise comparisons).

Overall, 91 samples were analyzed by HPLC-MS/MS. The median AqH concentration of ofloxacin in group I (control cohort) was 199.9 ng/mL (range 92.2–442.8 ng/mL), whereas the media of the non-severe DED patient group II was 530.5 ng/mL (range 283.7–1004.9 ng/mL), and the median of the severe DED patient group III was 719.2 ng/mL (range 358.0–1512.4 ng/mL). The Kruskal–Wallis test showed a significant difference in the ofloxacin levels in the AqH among the three groups (*p* < 0.001). Pairwise comparisons between groups (two-tailed, with a Bonferroni correction for multiple tests) resulted in a *p*-value of 0.001 when group I was compared to group II and group I was compared to group III, and *p*-values of 0.02 when group II was compared to group III (Figure 1).

**Figure 2:**
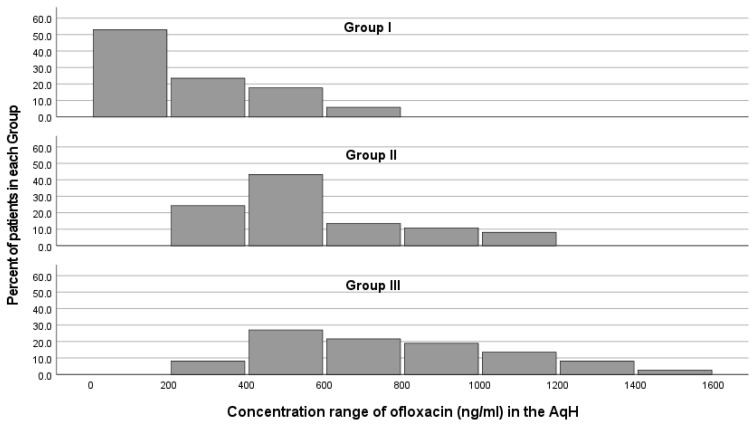
Histograms showing the concentration (ng/mL) range of ofloxacin in the AqH after topical drop instillation by the percentage of patients in each group. Group I, cataract patients without DED; group II, cataract patients with non-severe DED; group III, cataract patients with severe DED.

Histograms of the concentration range of ofloxacin among each group are presented in Figure 2. The concentration range of ofloxacin and the percentage of patients with higher concentrations of ofloxacin in the AqH are augmenting gradually from group I to groups II and III. In group I, almost half of the patients (52.9%) corresponded with an ofloxacin concentration of less than 200 ng/mL, and a quarter (23.5%) of patients had a concentration between 200 ng/mL and 400 ng/mL. In group II, most patients (43.2%) were found to have a concentration between 400 ng/mL and 600 ng/mL, 24.3% between 200 and 400 ng/mL, and 13.5% between 600 ng/mL and 800 ng/mL. Group III exhibited a wider concentration range (1189.45 ng/mL) compared to group II (820.83 ng/mL) and group I (616.76 ng/mL). A peak percent of patients (27%) was found to be in the concentration range of 400 ng/mL to 600 ng/mL, followed by 21.6% of patients between 600 ng/mL and 800 ng/mL, 18.9% between 800 ng/mL and 1000 ng/mL, and 13.5% between 1000 ng/mL and 1200 ng/mL. The correlation study between the severity of DED across group I (cataract patients without DED), group II (mild/moderate DED), group III (severe DED), and the levels of ofloxacin revealed a strong positive correlation (r = 0.639, *p* < 0.001).

## 4. Discussion

DED has globally evolved into a public health concern (it affects 6–34% of the global adult population), and it shows increasing prevalence worldwide [26,27]. It is recognized as being among the most common ocular conditions to seek treatment for [28]. Moreover, DED is a common comorbidity in ocular diseases usually treated by topical drops, such as glaucoma, the most common cause of blindness, Sjogren’s syndrome, and graft versus host disease [27,29,30]. Based on the disease’s prevalence, the effect of DED on the bioavailability of a topically applied drug in the A/C is plausible. None of the patients participating in the study developed post-operative endophthalmitis or any other ocular infection after cataract surgery.

To the best of our knowledge, the current study is the first to evaluate drug concentration in the AqH after topical instillation in patients with DED. Our results confirm that the disease’s severity positively correlates with drug concentration in the AqH (Figure 1). The median determined concentration values of ofloxacin augmented gradually from group I through to groups II and III (Figure 2). Compared to the control group, median levels of ofloxacin were increased almost two-fold in group II and increased three-fold in group III (*p* < 0.001).

The topical application of antibiotics has considerable advantages. Confined systemic exposure leads to reduced risk of side effects that may result from administration through other common routes (per os, intravitreally), reduced risk of resistance, and the achievement of high concentrations on the ocular surface. Bouchard et al. reported that AqH aspiration within one hour of the last ofloxacin dose led to diminished AqH concentration in comparison to sampling afterward [31]. Nevertheless, the cornea comprises a complicated barrier in intraocular penetration, mainly subject to its unique anatomy and the physicochemical properties of the antimicrobial agent. Drugs need to be both lipid- and water-soluble. Furthermore, elimination through the conjunctival vasculature and the tear drainage system rapidly reduces the tear concentration of the instilled drug, rendering the frequency of the applications a key element of bioavailability [9,10,12,32,33].

Apart from these major ocular pharmacokinetic factors, drugs are usually administered under disease conditions that may differ from those normally present [34]. The impact of inflammation has been extensively discussed, due to the physiological (e.g., lymphangiogenesis, increased vascular permeability) alterations and the possible damage inflicted on tissues [35,36,37]. DED is considered an inflammatory disease, originating from tear hyperosmolarity and becoming amplified by the progression of numerous conditions (systemic diseases, such as Sjogren’s disease or rheumatoid arthritis; meibomian gland dysfunction; blepharitis; environmental factors, such as intense UV exposure or low humidity; toxic effects, such as preservatives of topical drugs; allergies; contact lens use; other concomitant eye inflammations, such as viral/bacterial conjunctivitis, ocular surgeries, hormonal imbalance, lid margin irregularities, or increased oxidative stress), resulting in the progressive destruction of the ocular surface and patient discomfort in accordance with disease progression. The disruption of corneal barriers may promote intraocular drug penetration, but conjunctival epithelium squamous metaplasia, in conjunction with the lymphangiogenesis in both the conjunctiva and cornea, may counteract this [2,3,5,38,39,40,41,42]. Animal in vivo studies, after the topical instillation of ofloxacin, confirmed higher intraocular bioavailability in inflamed eyes (mean AqH concentrations were significantly augmented), though this was due to intraocular infection rather than ocular surface inflammation [34,36,37].

The ofloxacin concentrations determined in the AqH within the three cohorts of our study were compared with ofloxacin’s median MIC_90_ towards the main pathogens causing endophthalmitis [43,44] (Table 1). The median ofloxacin concentration of 199.9 ng/mL measured in patients without DED (group I) seems to be adequate only against *gram-negative bacteria*. However, in 23.5% of the group I patients, ofloxacin levels were measured in the AqH to be above the MIC_90_ of *coagulase-negative Staphylococci* and *Bacillus* sp., whereas the ofloxacin levels were above the MIC_90_ of *Staphylococcus aureus* in only 5.9% of the group I patients. In contrast, in patient groups II and III (with non-severe and severe DED, respectively), ofloxacin concentrations were above the MIC_90_ for *gram-negative bacteria*. Further, in the majority of group II and III patients (78.4% and 94.6%, correspondingly), ofloxacin concentrations were found to be above the MIC_90_ of *coagulase-negative Staphylococci* and *Bacillus* sp. In 27.0% and 54.1% of patients in groups II and III, respectively, levels of ofloxacin were found to be above the MIC_90_ of *S. aureus*. A noteworthy finding is that 2.7% of the group III patients showed ofloxacin levels that were effective against *β-hemolytic Streptococcus*. None of the DED or no-DED patients had ofloxacin levels that measured above the MIC_90_ against *Enterococci* sp., *Streptococcus viridans,* or *Streptococcus pneumoniae*. The current topical ofloxacin instillation scheme might be inefficient in the prevention of post-operative endophthalmitis caused by a considerable number of common endophthalmitis pathogens.

The Current study with DED patients confirms the positive impact of inflammation in ocular therapeutics, as it may enhance the bioavailability of topical formulations, which is linked to more effective treatment of ocular diseases. This has also been shown in cases of other ocular inflammatory diseases (diabetes [45], proliferative vitreoretinopathy, and penetrating eye injury with an intraocular foreign body [46]). In order to evaluate the impact of ocular surface inflammation on ocular pharmacokinetics after topical drop instillation, the steady state and maximum concentration of every drug should be determined by implementing several different time points of sample collection.

## 5. Conclusions

According to the finding of the current study, ofloxacin concentration in the A/C after topical drop instillation is affected by the degree of ocular surface inflammation in patients suffering from DED. Further studies are needed to confirm the impact of DED on ocular pharmacokinetics, considering the high prevalence of the disease.

## Figures and Tables

**Table 1:** Median minimum inhibitory concentrations (MIC_90_) of ofloxacin in the AqH for common endophthalmitis pathogens [43].

Organism	Ofloxacin MIC_90_ (ng/mL)
*Streptococcus pneumoniae*	2000
*Streptococcus viridans*	2000
*Enterococci species*	2000
*β-hemolytic Streptococcus*	1500
*Staphylococcus aureus*	630
*Bacillus species*	380
*coagulase-negative Staphylococci*	380
*gram-negative bacteria*	190

## Data Availability

The data presented in this study are available upon reasonable request to the corresponding author.
